# Synaptic and Intrinsic Activation of GABAergic Neurons in the Cardiorespiratory Brainstem Network

**DOI:** 10.1371/journal.pone.0036459

**Published:** 2012-05-03

**Authors:** Julie G. Frank, David Mendelowitz

**Affiliations:** Department of Pharmacology and Physiology, The George Washington University, Washington, DC, United States of America; Yale School of Medicine, United States of America

## Abstract

GABAergic pathways in the brainstem play an essential role in respiratory rhythmogenesis and interactions between the respiratory and cardiovascular neuronal control networks. However, little is known about the identity and function of these GABAergic inhibitory neurons and what determines their activity. In this study we have identified a population of GABAergic neurons in the ventrolateral medulla that receive increased excitatory post-synaptic potentials during inspiration, but also have spontaneous firing in the absence of synaptic input. Using transgenic mice that express GFP under the control of the *Gad1* (GAD67) gene promoter, we determined that this population of GABAergic neurons is in close apposition to cardioinhibitory parasympathetic cardiac neurons in the nucleus ambiguus (NA). These neurons fire in synchronization with inspiratory activity. Although they receive excitatory glutamatergic synaptic inputs during inspiration, this excitatory neurotransmission was not altered by blocking nicotinic receptors, and many of these GABAergic neurons continue to fire after synaptic blockade. The spontaneous firing in these GABAergic neurons was not altered by the voltage-gated calcium channel blocker cadmium chloride that blocks both neurotransmission to these neurons and voltage-gated Ca^2+^ currents, but spontaneous firing was diminished by riluzole, demonstrating a role of persistent sodium channels in the spontaneous firing in these cardiorespiratory GABAergic neurons that possess a pacemaker phenotype. The spontaneously firing GABAergic neurons identified in this study that increase their activity during inspiration would support respiratory rhythm generation if they acted primarily to inhibit post-inspiratory neurons and thereby release inspiration neurons to increase their activity. This population of inspiratory-modulated GABAergic neurons could also play a role in inhibiting neurons that are most active during expiration and provide a framework for respiratory sinus arrhythmia as there is an increase in heart rate during inspiration that occurs via inhibition of premotor parasympathetic cardioinhibitory neurons in the NA during inspiration.

## Introduction

Rhythmically active neuronal networks are critically involved in numerous physiological and cognitive functions and are essential for various behaviors such as sleep, addiction, arousal, memory and breathing. The formation of spatial memory is reliant on theta rhythm in the hippocampus, for example [1]. Pacemaker neurons are key rhythmicity generators in many of these networks [2], [3]. The most well studied pacemaker network exists in the suprachiasmatic nucleus of the hypothalamus, an area that is responsible for food intake, sleep and the regulation of body temperature and heart rate [3], [4]. Another example of pacemaker modulation of neural networks is in the respiratory system; it has been postulated that the preBötzinger complex (preBötC) is the site of respiratory rhythm generation [5], [6].

The neurotransmitter GABA is known to play a vital function in several pacemaker networks, but little is known regarding the role of GABAergic neurons as a direct source or modulator of inspiratory activity in the respiratory network. GABAergic neurons have been found in several brain regions involved in the control of cardiorespiratory function [7]–[10] including the nucleus tractus solitarius (NTS) [11], [12] and the ventrolateral medulla [13], [14]. The modulation of heart rate and production of respiratory sinus arrhythmia (RSA) [15], [16] is dependent upon both the activity of the cardioinhibitory parasympathetic system, originating from brainstem cardiac vagal neurons (CVNs), and the GABAergic neurons active in inspiration that project to and inhibit CVNs during each inspiration. Diminished CVN activity and RSA are strong risks factors and predictors of morbidity and mortality [17], [18].

Recent work has shown that four distinct areas in the brainstem are the origin of GABAergic neurons that project to CVNs, three in the vicinity of the nucleus ambiguus (NA), corresponding to the rostro-ventral lateral medulla (RVLM) and preBötC, and one in the NTS [19]. Similarly, inspiratory-modulated GABAergic neurons have been localized to sites within the ventral medulla: Kuwana et al. demonstrated a population of GABAergic neurons in the preBötC that fire bursts of action potentials in unison with hypoglossal rootlet firing, but did not characterize the firing properties or synaptic inputs to these neurons [20]. In this study we tested two hypotheses relevant to a fundamental issue in the origin of rhythmogenesis within the cardiorespiratory network: (1) the subpopulation of GABAergic neurons in these distinct brainstem sites depends upon increased excitatory glutamatergic and/or cholinergic neurotransmission during inspiration to generate respiratory related activity; or (2) they possess inherent respiratory pacemaker like properties that enable them to fire spontaneously and aid the initiation of respiratory patterning. To test these hypotheses, we identified a population of GABAergic neurons in the brainstem via expression of GFP under the control of the *Gad1* (GAD67) gene promoter, and examined the role of excitatory synaptic neurotransmission and inherent firing properties of these neurons in both voltage clamp and current clamp conditions.

## Methods

### Subjects

All animal procedures were performed with the approval of the Animal Care and Use Committee of The George Washington University in accordance with the recommendations of the panel on euthanasia of the American Veterinary Medical Association and the National Institutes of Health *Guide for the Care and Use of Laboratory Animals*.

GABAergic neurons were visualized from transgenic mice (Jackson Laboratories, Bar Harbor, MA) expressing GFP under the control of the *Gad1* (GAD67) gene promoter. Pups were 3–7 days old on the day of the experiment. All mice were maintained in a 12-hour light/dark cycle.

### Procedures

CVNs were retrogradely labeled from the parasympathetic ganglia in the fat pads at the base of the heart as described previously [21]. In brief, pups were exposed to hypothermia to slow heart rate. The heart was exposed by a right thoractomy and the retrograde fluorescent tracer X-rhodamine-5-(and 6)-isothiocyanate (Molecular Probes, Eugene, OR) was injected into the fat pads at the base of the heart within the pericardial sac. The fluorescent tracer was absorbed by the terminals of the preganglionic parasympathetic neurons and then retrogradely transported to the cell bodies of CVNs in the NA. After at least 24 hours recovery pups were anesthetized with isoflurane, sacrificed by cervical dislocation, and the hindbrain was removed and placed in cold physiological saline solution (in mM: 140 NaCl, 5 KCl, 2 CaCl_2_, 5 glucose, 10 HEPES, bubbled with 100% O_2_, pH 7.4).

### Preparation

**Figure 1:**
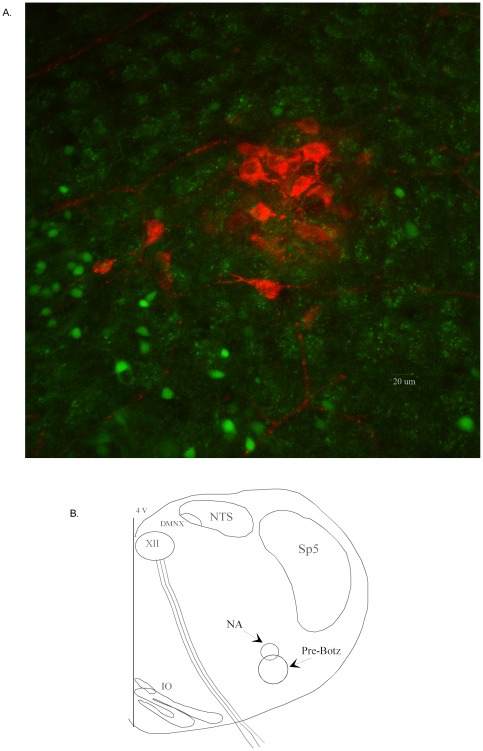
Images of GABAergic neurons in proximity to CVNs. a. In this confocal image, CVNs in the NA are labeled with rhodamine and are surrounded by smaller GABAergic cell bodies and terminals labeled with GFP. b. The 800 µm thick transverse medullary slice preparation from P3–P6 mice has all the necessary circuitry for respiratory rhythm generation including the preBötC, respiratory hypoglossal motorneurons and intact hypoglossal nerve rootlets, and the NA.

The medulla was mounted on a wax cutting block and placed in a vibrating blade microtome (Leica, Nussloch, Germany). Serial transverse sections were sliced in rostro-caudal progression until the inferior olives and the NA could be visualized on the rostral surface of the tissue. An 800 µm thick slice was taken, containing the parasympathetic CVNs, the rostral hypoglossal nucleus and rootlets, and the preBötC (Figure 1) and additional circuitry for respiratory activity [6]. Spontaneous and respiratory-related activity was recorded by monitoring motor-neuron population activity from hypoglossal nerve rootlets using a suction electrode. Hypoglossal rootlet activity was amplified 50,000 times, was filtered (10- to 300-Hz band pass; CWE Inc., Ardmore, PA) and electronically integrated (τ = 50 ms; CWE Inc.).

### Confocal Microscopy

Confocal images were collected on a Zeiss LSM 710 system (Carl Zeiss Microimaging GmbH), equipped with Axio Examiner Z1 upright microscope and W Plan-Apo 20× (NA, 1.0) (DIC VIS-IR WD = 1.8) and Plan-Apochromat 63× (NA, 1.40) oil (DIC) objectives, was used. The system has a 32 channel spectral-detection Quasar photomultiplier and two single channel photomultipliers to record the backward emission. Argon 488 line of a multiline 25 mW argon laser was used to excite GFP, whereas rhodamine was excited with 5 mW HeNe emitting at 633 nm. The microscope was equipped with Prior x/y/z scanning stage, which permitted capturing tile stack images. Emission filtering was adjusted by setting the desired spectral window for recording using the Zen 2009 software. In addition, the Zen 2009 software provided an online, linear spectral unmixing algorithm, which allowed the separation of several dyes based on spectral characteristics, despite emission spectra overlap, known as online spectral fingerprinting. Emission filtering was adjusted by setting the desired spectral window for recording.

### Whole Cell Patch-Clamp

Slices were perfused in room temperature aCSF (in mM: 125 NaCl, 3 KCl, 2 CaCl_2_, 26 NaHCO_3_, 5 glucose, 5 HEPES, equilibrated with 95% O_2_, 5% CO_2_, pH 7.35–7.4). Patch pipettes (2.5–3.5 MΏ) containing a potassium gluconate solution of the following composition (in mM): 135 K+ gluconic acid, 10 HEPES, 10 EGTA, 1 CaCl_2_, and 1 MgCl_2_, pH 7.4 were visually guided to the surface of individual GABAergic neurons using differential interference optics and infrared illumination (Zeiss, Oberkochen, Germany). The pipette was advanced until a seal was obtained over 1 GΩ between the pipette tip and the cell membrane of the identified neuron. The membrane under the pipette tip was then ruptured with a brief suction to obtain whole cell patch-clamp recordings. All the drugs used in these experiments were either focally applied to the patched neuron using a pneumatic picopump pressure delivery system and were continuously ejected from a glass micropipette positioned within 30 µm from the patched neuron (pressure <4 psi), or were included in the perfusate. The maximum range of drug application with focal application has been determined previously to be 100–120 µm downstream from the drug pipette and considerably less behind the drug pipette [22]. EPSCs were isolated by the focal application of gabazine (25 µM) and strychnine (1 µM) to block GABAergic and glycinergic postsynaptic currents, respectively, throughout the experiment. Dihydro-beta-erythroidine (DHßE) at 1 µM and 100 µM, and alpha-bungarotoxin (α-Btx) at 100 nM were focally applied to block α4ß2 and α7 nicotinic receptor activity, respectively. All synaptic events were blocked at the end of each experiment with the focal application of D-2-amino-5-phosphonovalerate (AP-5, 50 µM), and 6-cyano-7-nitroquinoxaline-2,3-dione (CNQX, 50 µM) to block NMDA and non-NMDA glutamatergic receptors, respectively.

For current clamp experiments, CNQX (50 µM) and AP-5 (50 µM) were added to the perfusate to block all glutamatergic neurotransmission within the tissue to examine potential the pacemaker properties of these neurons. 8 out of 20 cells continued to fire in the presence of these glutamatergic antagonists and were examined further for pacemaker properties by inclusion of 100 µM cadmium chloride or 20 µM riluzole in the perfusate.

### Statistical Analyses

Results are graphically presented as means ± SE. EPSC frequency was grouped into 1-s bins and cross-correlated with the onset of inspiratory–related hypoglossal activity. Inspiratory bursts were identified from the raw hypoglossal output, and only bursts whose amplitude was double the basal noise were analyzed. Statistical comparisons of burst related activity were performed using one-way ANOVA and Neumann-Keuls for post hoc analysis, comparisons before and after drug applications without bursting were compared using paired T-tests. Statistical significance for all data was set at p<0.05.

## Results

As shown in Figure 1a, parasympathetic cardiac neurons, identified by the presence of the fluorescent tracer rhodamine (shown in red) [23] and smaller GABAergic neurons (shown in green) identified by visualization of the expressed GFP, were co-localized in the ventrolateral medulla. GABAergic cell bodies were located in close proximity to CVNs, and numerous GABAergic punctuate axonal fibers and contacts made close apposition to, and in some examples partially encircled, the somata of CVNs.

To identify respiratory related GABAergic neurons we utilized a thick *in vitro* brainstem slice for whole cell recordings from GABAergic neurons while simultaneously recording inspiratory activity from the hypoglossal rootlet. As previous work [19] identified 4 specific foci as the origin of GABAergic neurons that project to CVNs we limited our study to those GABAergic neurons directly ventral to the NA as most likely involved in cardiorespiratory interactions (see Figure 1b). Approximately 1 in 20 GABAergic neurons in this area displayed respiratory related activity, defined as possessing either a 25 percent increase in EPSCs while recorded in voltage clamp configuration, or having increased spontaneous firing during inspiratory activity, recorded in current clamp configuration, and subsequent experiments were limited to this population of respiratory related GABAergic neurons.

As previous work has shown focal application of the nicotinic receptor antagonist dihydro-beta-erythroidine (in a beta2-selective concentration, 3 µM) abolished the respiratory-evoked increase in GABAergic neurotransmission to CVNs [16], we hypothesized nicotinic cholinergic receptors would play a role in the function and activity of these inspiratory active GABAergic neurons. We used two different nicotinic receptor antagonists, DHßE and α-Btx. DHßE has been shown to block nicotinic receptors containing ß2 subunits at lower concentrations (1 µM–3 µM) and all heteromeric receptors at higher concentrations (50–100 µM) [24], [25], while α-Btx blocks homomeric α7 nicotinic receptors [26].

**Figure 2:**
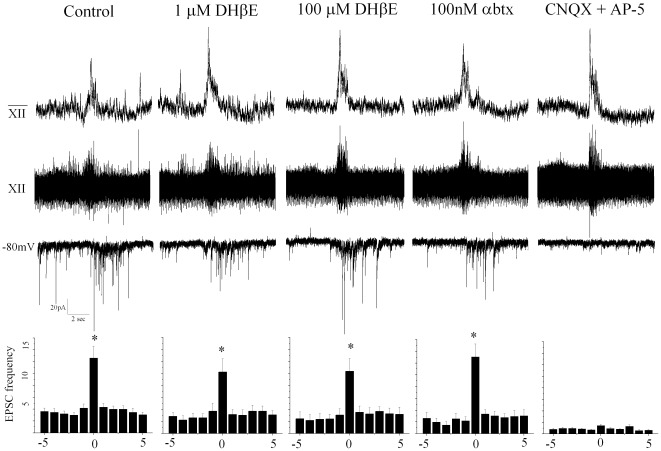
Respiratory-related GABAergic neurons receive glutamatergic input that is blocked by antagonists CNQX and AP-5. Glutamatergic receptors mediate excitatory neurotransmission to inspiratory GABAergic neurons. Inspiratory-related bursting activity was recorded from the hypoglossal rootlet (top trace) and electronically integrated (middle trace). Excitatory transmission to GABAergic neurons (bottom trace) was isolated by focal application of GABA (gabazine; 25 µM) and glycine (strychnine; 1 µM) receptor antagonists. Under control conditions there was a significant increase in EPSC frequency in the identified GABAergic neurons during inspiratory bursts control spontaneous: 3.5+/−0.5, control inspiratory: 12.6+/−1.9 Hz. DHßE (at concentrations of 1 µM, n = 8 and 100 µM, n = 7), and α-Btx (100 nM, n = 6) did not significantly change spontaneous or inspiratory related EPSC frequency. CNQX and AP5 blocked EPSC spontaneous and inspiratory frequency (p<0.05). The average results from all neurons tested (DHßE at 1 µM n = 8, at 100 µM n = 7, and with α-Btx at 100 nM, n = 6) are shown in the peri-inspiratory activity histogram, bottom, illustrating the average EPSC frequency (Hz) for each of the 5 seconds before the burst, frequency during the inspiratory burst (which typically lasted 1–2 seconds), and each of the 5 seconds following the inspiratory burst.

Neither the low (n = 8) or high (n = 7) concentration of DHßE had any significant effect on spontaneous or inspiratory EPSCs in these GABAergic neurons, as shown in figure 2 (control spontaneous: 3.5+/−0.5, control inspiratory: 12.6+/−1.9 Hz, 1 µM: spontaneous: 2.8+/−0.8 Hz; inspiratory: 10.2+/−2.2 Hz; 100 µM: spontaneous: 2.5+/−0.9 Hz; inspiratory: 10.3+/−2.1 Hz). Similarly, 100 nM α-Btx did not alter either spontaneous or inspiratory-related synaptic neurotransmission to GABAergic neurons (n = 6, control spontaneous: 3.6+/−1.0, inspiratory 11.7+/−1.5 Hz, 100 nM α-Btx spontaneous: 2.2+/−0.8 Hz; inspiratory: 13.1+/−2.2 Hz). All synaptic activity to these GABAergic neurons was glutamatergic as shown by the abolishment of EPSCs with the AMPA/kainate and NMDA receptor antagonists CNQX and AP-5, respectively (n = 7).

**Figure 3:**
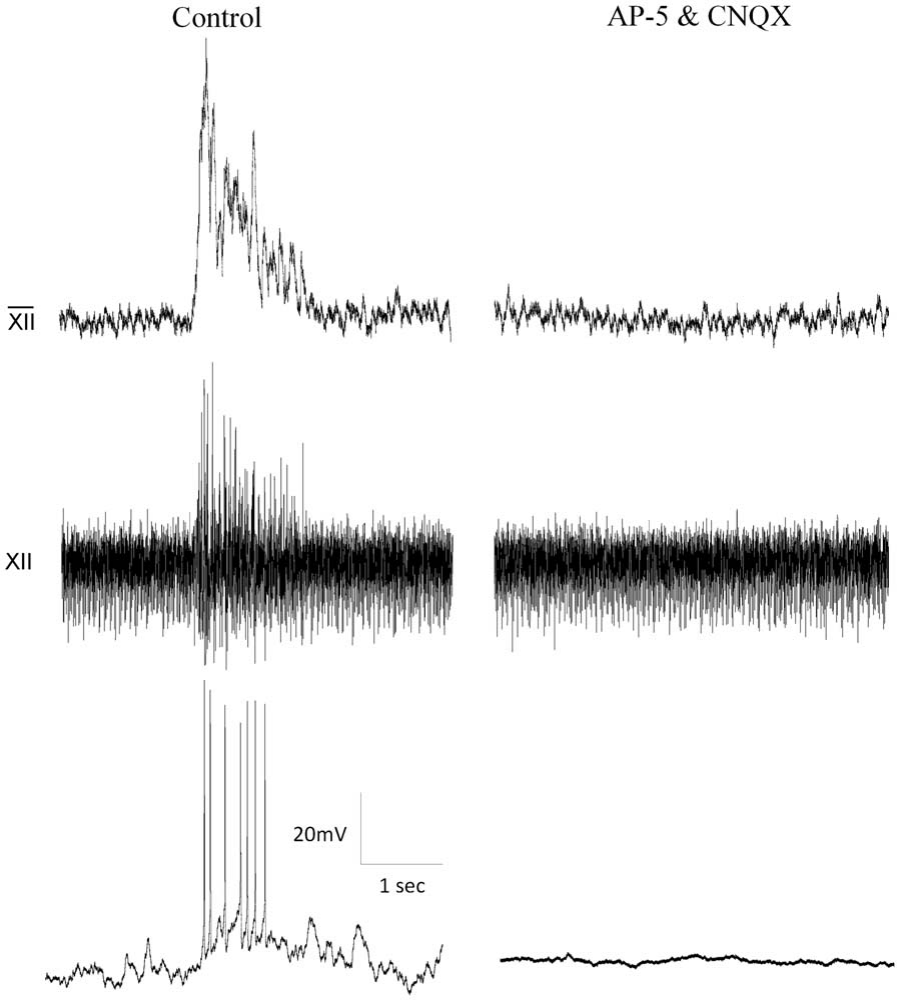
GABAergic inspiratory nonpacemaker cells fire in bursts that are blocked by CNQX and AP-5. GABAergic neurons fire bursts of action potentials during inspiration. Cells were patch clamped in the current clamp configuration with a small amount of current when needed (<30 pA) injected to hold it at −65 mV. Inspiratory activity was measured from the hypoglossal rootlets (top two traces). Respiratory activity was greatly reduced in this population of cells. (Control: 1.7+/−0.4 Hz; CNQX & AP5: 0.2+/−0.3 Hz; p<0.05).

**Figure 4:**
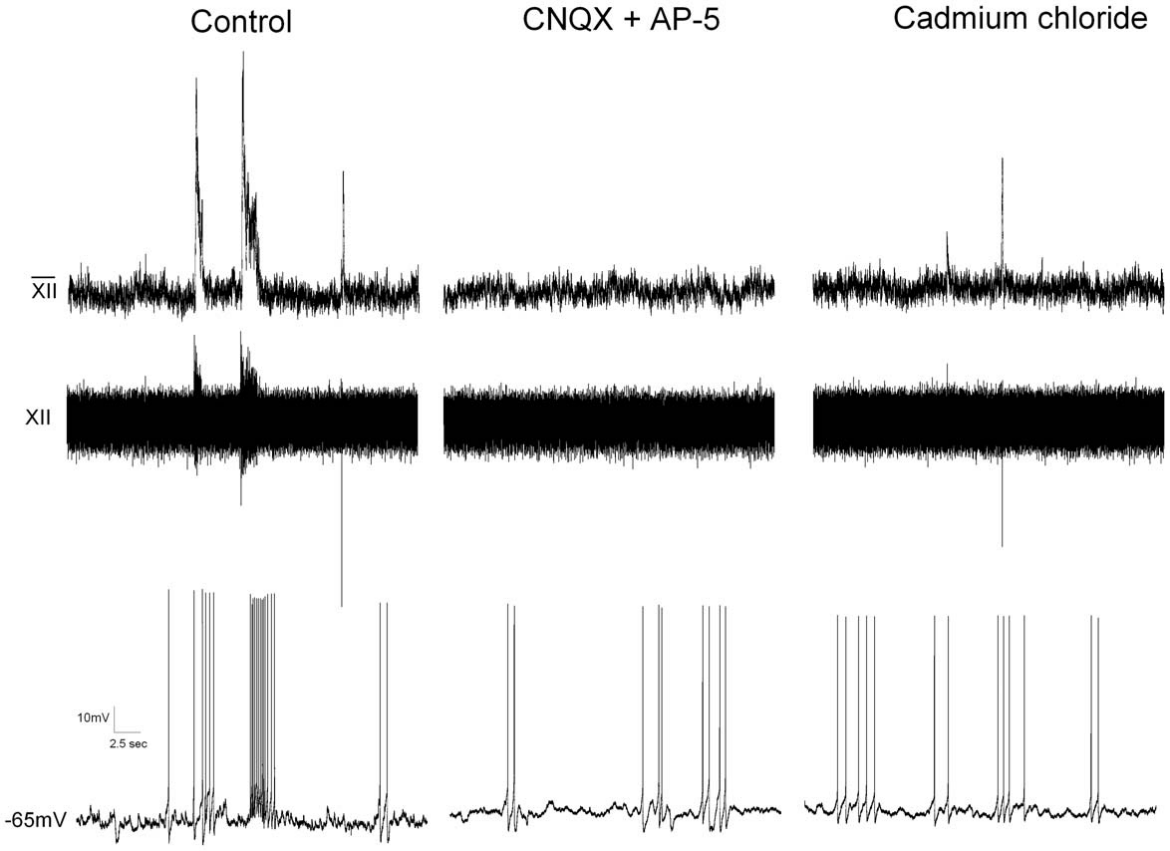
Firing in GABAergic pacemaker neurons is altered but not blocked by glutamatergic antagonists. GABAergic neurons fire bursts of action potentials during XII bursts. Cells were patch clamped in the current clamp configuration with a small amount of current (<30 pA) injected to hold the cell at −65 mV. Inspiratory activity was measured from the hypoglossal rootlets (top two traces). Fewer than half of these cells (n = 8/20) continued to fire in the presence of CNQX and AP-5. In cells that showed continued activity, the synaptic activity to these neurons, as well as voltage gated calcium channels, were blocked by addition of cadmium chloride. Pacemaker activity patterns changed but frequencies between the three groups was not significantly different (Control: 1.7+/−0.4 Hz; CNQX & AP5: 1.7+/−0.5 Hz; CdCl_2_: 1.8+/−0.6 Hz).

To examine the spontaneous pacemaker-like properties and firing patterns of inspiratory active GABAergic neurons these cells were recorded in the current clamp configuration. These cells fired bursts of action potentials that started with the onset of inspiratory activity in the hypoglossal rootlet; spontaneous firing frequency increased from 1.4+/−0.5 prior to the inspiratory burst to 5.9+/−1.0 Hz during inspiratory activity, (n = 20). To determine whether these action potentials were being generated by synaptic inputs or were due to intrinsic pacemaker-like properties of the cell, the glutamatergic antagonists CNQX (50 µM) and AP-5 (50 µM) were included in the perfusate. In greater than half of the examined GABAergic neurons (12 of 20), the glutamate receptor antagonists abolished all spontaneous activity (Figure 3). In the remaining 8 GABAergic neurons (out of 20 total) that continued to fire in the presence of these glutamatergic receptor antagonists, the overall frequency of action potential firing was not different in the presence of the glutamatergic receptor antagonists (control: 1.7+/−0.4 Hz; CNQX & AP-5: 1.7+/−0.5 Hz, n = 8), see Figure 4.

To determine if spontaneous activity in these neurons could occur in the absence of all synaptic inputs to these neurons the calcium channel blocker cadmium chloride (100 µM) was added to the perfusate to prevent synaptic release of transmitters. The spontaneous firing of GABAergic neurons in this study was not altered by the inclusion of cadmium chloride (control frequency = 1.7+/−0.4, with AP-5 and CNQX 1.7+/−0.5 and in the presence of CdCl_2_ 1.8+/−0.6 Hz, n = 8, p>0.05), indicating this GABAergic population of pacemaker neurons continue to fire in the absence of neurotransmitter release and synaptic activity. However the pattern of firing became more variable in the presence of AP-5 and CNQX, as well as with CdCl_2_, and as a measure of this variability the coefficient of variance increased from a control value of 0.67 to 0.82 in the presence of AP-5 and CNQX, and 0.94 with CdCl_2._

**Figure 5:**
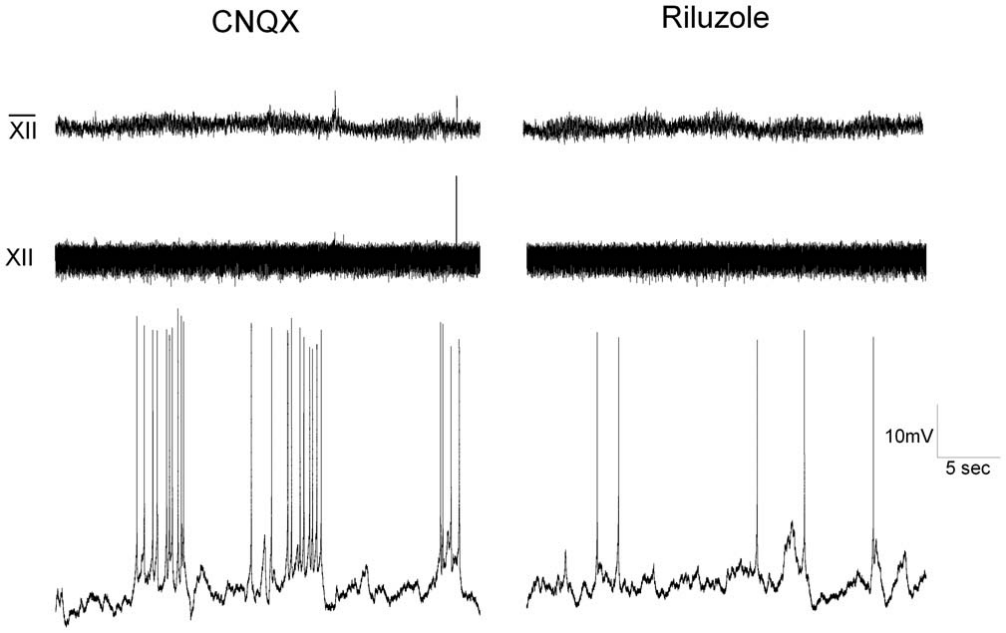
GABAergic pacemaker activity is reduced by the persistent sodium current blocker riluzole. GABAergic neurons were patch clamped in the current clamp configuration. GABAergic neurons fire bursts of action potentials even in the presence of excitatory glutamatergic receptor blockers AP-5 and CNQX. However, application of 20 µM riluzole, the persistent sodium channel blocker nearly abolished all activity (p<0.05) (CNQX: 2.9+/−0.8 Hz; riluzole: 1.1+/−0.3, n = 6).

In an additional set of experiments (Figure 5), the role of the persistent sodium current (I_NaP_) was examined by bath application of 20 µM riluzole, an I_NaP_ blocker. Riluzole significantly (p<0.05) reduced, but did not abolish, spontaneous action potential firing (prior to riluzole: 2.9+/−0.8 Hz; riluzole: 1.1+/−0.3 Hz, n = 6), coefficient of variance 0.67.

## Discussion

Discovering the neurons and neurotransmitters underlying respiratory genesis and function is vital for our understanding of cardio-respiratory control in the brainstem, both in healthy and disease states. In this study, using a fictive respiratory brainstem preparation, we identified a population of inspiratory GABAergic neurons in the ventral medulla that receive bursts of excitatory neurotransmission during inspiration. This excitatory transmission was abolished with the glutamatergic receptor antagonists AP-5 and CNQX. In addition, a subset of these inspiratory GABAergic neurons continued to fire action potentials in the absence of excitatory synaptic drive, indicating these GABAergic neurons may possess pacemaker-like properties. Moreover, the spontaneous firing in these GABAergic neurons was not abolished by blocking all synaptic inputs to these neurons using the voltage-gated calcium channel blocker cadmium chloride, indicating that putative pacemaker activity in these neurons is independent of both synaptic input and voltage gated calcium channels. However spontaneous firing was significantly diminished by the persistent sodium channel blocker riluzole, demonstrating a role of this channel in the pacemaker-like activity of the cardiorespiratory GABAergic neurons. However while these neurons continued to fire in the absence of synaptic neurotransmission, their firing was more sporadic, suggesting even with intact intrinsic membrane properties that are sufficient for firing, neurotransmission plays an important role in generating bursts of activity in these neurons.

Pacemaker cells have been shown to express a persistent sodium current (I_NaP_) [6], [27], [28] and/or voltage activated calcium current (ICa) and both of these channels can facilitate rhythmogenesis [24]. Cadmium-insensitive pacemakers rely on the I_NaP_, whereas cadmium-sensitive pacemakers depend on the activation of calcium currents. I_NaP_ is ubiquitous in the preBötC throughout all developmental periods and is hypothesized to play a critical role in rhythm generation because of its subthreshold activation and slow inactivation properties [29]. In this study we demonstrate I_NaP_ plays a role in the continued activity of these GABAergic inspiratory neurons as evidenced by the significant decrease in frequency seen with riluzole. In contrast, there are developmental changes in the function of the ICa channel: a recent study showed little to no response to cadmium chloride in pacemaker neurons from mice ages P0–P5, while in mice ages P8–P10 7.5% of pacemaker cells responded with a decrease in bursting [30]. In this study GABAergic neurons continued to fire after blockade of glutamatergic neurotransmission with glutamate receptor antagonists, as well as during complete synaptic inhibition by application of cadmium. Since our studies were conducted in mice between P3–P6, we cannot rule out the possibility that the ICa channel plays a role in these GABAergic neurons later in development, or alternatively, these GABAergic pacemaker cells may not express these channels at any point during development and instead depend fully upon the I_NaP_, for the generation of respiratory pacemaker activity.

In this study we hypothesized nicotinic cholinergic receptors would facilitate the excitatory neurotransmission to respiratory modulated GABAergic neurons by increasing the release of glutamate. This hypothesis was based upon previous studies from our lab and others that have shown nicotinic acetylcholine receptors modulate respiratory activity in the brainstem [31], [32] and facilitate GABAergic synaptic transmission to CVNs during the respiratory cycle [16], [33]. However, nicotinic receptor activation played no endogenous role in the excitatory synaptic neurotransmission to these GABAergic neurons as blocking nicotinic receptors did not alter any aspect of the excitatory neurotransmission to these GABAergic neurons during either quiescence or rhythmic inspiratory activity. The ß4, ß2, and α7 nicotinic subunits are all present in the brainstem in various regions including those involved in the generation and maintenance of respiration [7], [34]. One possible explanation for this surprising finding of a lack of cholinergic regulation on excitatory neurotransmission to GABAergic neurons is that nicotinic receptors are present and are essential for the synaptic release of GABA from GABAergic synaptic terminals, but nicotinic receptors are not localized and/or involved in postsynaptic activation occurring in the dendrites and soma of these GABAergic neurons, as well as in the preceding glutamatergic neurons that project to these GABAergic neurons.

Traditionally respiratory rhythmogensis is thought to involve only excitatory neurotranasmission for initiation; originating from either an isolated homogenous excitatory pacemaker population, or a network of coupled excitatory and spontaneously firing neurons with conditional oscillatory bursting properties that set the respiratory rhythm within the central pattern generator of the brainstem [6], [35]–[38]. However Richter and colleagues have suggested that inhibitory neurons may also play an essential role in respiratory pattern generation as inspiratory bursting sprouts from the “release" of cell populations from postsynaptic inhibition [39]. Inspiration begins when inhibition is withdrawn from inspiratory neurons, indicating a dynamic balance between synaptic inhibition and excitatory intrinsic membrane potential oscillations [39]. The spontaneously firing GABAergic neurons identified in this study that increase their activity during inspiration would support respiratory rhythm generation if they acted primarily to inhibit post-inspiratory neurons and thereby release inspiration neurons to increase their activity. The population of inspiratory-modulated, but not spontaneously active, GABAergic neurons might play a role in inhibiting neurons that are most active during expiration and would also provide a framework for respiratory sinus arrhythmia as there is an increase in heart rate during inspiration that occurs via inhibition of premotor parasympathetic cardioinhibitory neurons in the NA during inspiration.

## References

[1] Wang XJ (2002). Pacemaker neurons for the theta rhythm and their synchronization in the septohippocampal reciprocal loop.. J Neurophysiol.

[2] Muller KJ, Tsechpenakis G, Homma R, Nicholls JG, Cohen LB (2009). Optical analysis of circuitry for respiratory rhythm in isolated brainstem of foetal mice.. Philos Trans R Soc Lond B Biol Sci.

[3] Welsh DK, Takahashi JS, Kay SA (2010). Suprachiasmatic nucleus: cell autonomy and network properties.. Annu Rev Physiol.

[4] Tosini G, Pozdeyev N, Sakamoto K, Iuvone PM (2008). The circadian clock system in the mammalian retina.. Bioessays.

[5] Koshiya N, Smith JC (1999). Neuronal pacemaker for breathing visualized in vitro.. Nature.

[6] Smith JC, Ellenberger HH, Ballanyi K, Richter DW, Feldman JL (1991). Pre-Botzinger complex: a brainstem region that may generate respiratory rhythm in mammals.. Science.

[7] Dehkordi O, Millis RM, Dennis GC, Jazini E, Williams C (2007). Expression of alpha-7 and alpha-4 nicotinic acetylcholine receptors by GABAergic neurons of rostral ventral medulla and caudal pons.. Brain Res.

[8] Ellenberger HH (1999). Distribution of bulbospinal gamma-aminobutyric acid-synthesizing neurons of the ventral respiratory group of the rat.. J Comp Neurol.

[9] Stornetta RL, Guyenet PG (1999). Distribution of glutamic acid decarboxylase mRNA-containing neurons in rat medulla projecting to thoracic spinal cord in relation to monoaminergic brainstem neurons.. J Comp Neurol.

[10] Stornetta RL, McQuiston TJ, Guyenet PG (2004). GABAergic and glycinergic presympathetic neurons of rat medulla oblongata identified by retrograde transport of pseudorabies virus and in situ hybridization.. J Comp Neurol.

[11] Neff RA, Mihalevich M, Mendelowitz D (1998). Stimulation of NTS activates NMDA and non-NMDA receptors in rat cardiac vagal neurons in the nucleus ambiguus.. Brain Res.

[12] Wang J, Irnaten M, Mendelowitz D (2001). Characteristics of spontaneous and evoked GABAergic synaptic currents in cardiac vagal neurons in rats.. Brain Res.

[13] Favero MT, Takakura AC, de Paula PM, Colombari E, Menani JV (2011). Chemosensory control by commissural nucleus of the solitary tract in rats.. Respir Physiol Neurobiol.

[14] Urbanski RW, Sapru HN (1988). Putative neurotransmitters involved in medullary cardiovascular regulation.. J Auton Nerv Syst.

[15] Loewy AD, Spyer KM (1990).

[16] Neff RA, Wang J, Baxi S, Evans C, Mendelowitz D (2003). Respiratory sinus arrhythmia: endogenous activation of nicotinic receptors mediates respiratory modulation of brainstem cardioinhibitory parasympathetic neurons.. Circ Res.

[17] Thayer JF, Yamamoto SS, Brosschot JF (2010). The relationship of autonomic imbalance, heart rate variability and cardiovascular disease risk factors.. Int J Cardiol.

[18] Weber CS, Thayer JF, Rudat M, Wirtz PH, Zimmermann-Viehoff F (2010). Low vagal tone is associated with impaired post stress recovery of cardiovascular, endocrine, and immune markers.. Eur J Appl Physiol.

[19] Frank JG, Jameson HS, Gorini C, Mendelowitz D (2009). Mapping and identification of GABAergic neurons in transgenic mice projecting to cardiac vagal neurons in the nucleus ambiguus using photo-uncaging.. J Neurophysiol.

[20] Kuwana S, Tsunekawa N, Yanagawa Y, Okada Y, Kuribayashi J (2006). Electrophysiological and morphological characteristics of GABAergic respiratory neurons in the mouse pre-Botzinger complex.. Eur J Neurosci.

[21] Mendelowitz D, Kunze DL (1991). Identification and dissociation of cardiovascular neurons from the medulla for patch clamp analysis.. Neurosci Lett.

[22] Wang J, Irnaten M, Venkatesan P, Evans C, Baxi S (2002). Synaptic activation of hypoglossal respiratory motorneurons during inspiration in rats.. Neurosci Lett.

[23] Mendelowitz D (1996). Firing properties of identified parasympathetic cardiac neurons in nucleus ambiguus.. Am J Physiol.

[24] Thoby-Brisson M, Ramirez JM (2001). Identification of two types of inspiratory pacemaker neurons in the isolated respiratory neural network of mice.. J Neurophysiol.

[25] Yakel JL (2010). Gating of nicotinic ACh receptors: latest insights into ligand binding and function.. J Physiol.

[26] Patrick J, Boulter J, Goldman D, Gardner P, Heinemann S (1987). Molecular biology of nicotinic acetylcholine receptors.. Ann N Y Acad Sci.

[27] Feldman JL, Smith JC (1989). Cellular mechanisms underlying modulation of breathing pattern in mammals.. Ann N Y Acad Sci.

[28] Johnson SM, Koshiya N, Smith JC (2001). Isolation of the kernel for respiratory rhythm generation in a novel preparation: the pre-Botzinger complex “island".. J Neurophysiol.

[29] Pace RW, Mackay DD, Feldman JL, Del Negro CA (2007). Role of persistent sodium current in mouse preBotzinger Complex neurons and respiratory rhythm generation.. J Physiol.

[30] Del Negro CA, Morgado-Valle C, Hayes JA, Mackay DD, Pace RW (2005). Sodium and calcium current-mediated pacemaker neurons and respiratory rhythm generation.. J Neurosci.

[31] Shao XM, Feldman JL (2001). Mechanisms underlying regulation of respiratory pattern by nicotine in preBotzinger complex.. J Neurophysiol.

[32] Shao XM, Feldman JL (2002). Pharmacology of nicotinic receptors in preBotzinger complex that mediate modulation of respiratory pattern.. J Neurophysiol.

[33] Neff RA, Humphrey J, Mihalevich M, Mendelowitz D (1998). Nicotine enhances presynaptic and postsynaptic glutamatergic neurotransmission to activate cardiac parasympathetic neurons.. Circ Res.

[34] Dehkordi O, Haxhiu MA, Millis RM, Dennis GC, Kc P (2004). Expression of alpha-7 nAChRs on spinal cord-brainstem neurons controlling inspiratory drive to the diaphragm.. Respir Physiol Neurobiol.

[35] Balis UJ, Morris KF, Koleski J, Lindsey BG (1994). Simulations of a ventrolateral medullary neural network for respiratory rhythmogenesis inferred from spike train cross-correlation.. Biol Cybern.

[36] Duffin J (1991). A model of respiratory rhythm generation.. Neuroreport.

[37] Ogilvie MD, Gottschalk A, Anders K, Richter DW, Pack AI (1992). A network model of respiratory rhythmogenesis.. Am J Physiol.

[38] Rybak IA, Paton JF, Schwaber JS (1997). Modeling neural mechanisms for genesis of respiratory rhythm and pattern. I. Models of respiratory neurons.. J Neurophysiol.

[39] Richter DW, Ballanyi K, Schwarzacher S (1992). Mechanisms of respiratory rhythm generation.. Curr Opin Neurobiol.

